# Supplementary material for: A Three-Dimensional Nickel(II) Framework from a Semi-Flexible Bipyrimidyl Ligand Showing Weak Ferromagnetic Behavior
Source: Polymers (Basel). 2019 Jan 11;11(1):119. doi: 10.3390/polym11010119 (PMC6401916; doi:10.3390/polym11010119)
Supplement: Supplementary file 1 [file polymers-11-00119-s001.pdf]

A three-dimensional nickel(II) framework from a semi-flexible bipyrimidyl ligand showing weak ferromagnetic behavior

Shin-Shan Dong and Chen-I Yang*

Department of Chemistry, Tunghai University, Taichung 407, Taiwan

	Index	Page
**Figure S1.**	Simulated PXRD pattern (red) and experimental PXRD pattern of compound.	S2
**Figure S2.**	Thermogravimetric (TG) analysis diagram of compound **1**.	S3
**Figure S3.**	Plot of χ_M (○) *vs.* *T* for a powdered sample of compound **1**.	S4
**Figure S4.**	Plot of χ_M^{-1} (○) *vs.* T for a powdered sample of compound **1**. The solid line represents the best fit χ_M^{-1} above 50 K with a Curie–Weiss law.	S5
**Figure S5.**	EPR spectra of a powdered compound **1** collected at 77 K.	S6

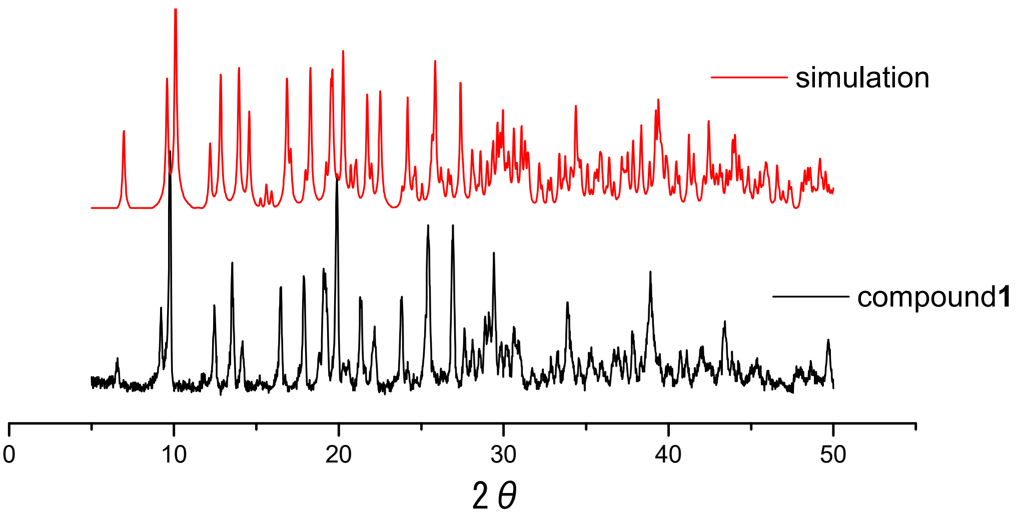

Figure. S1. Simulated PXRD pattern (red) and experimental PXRD pattern (black) of compound **1**.

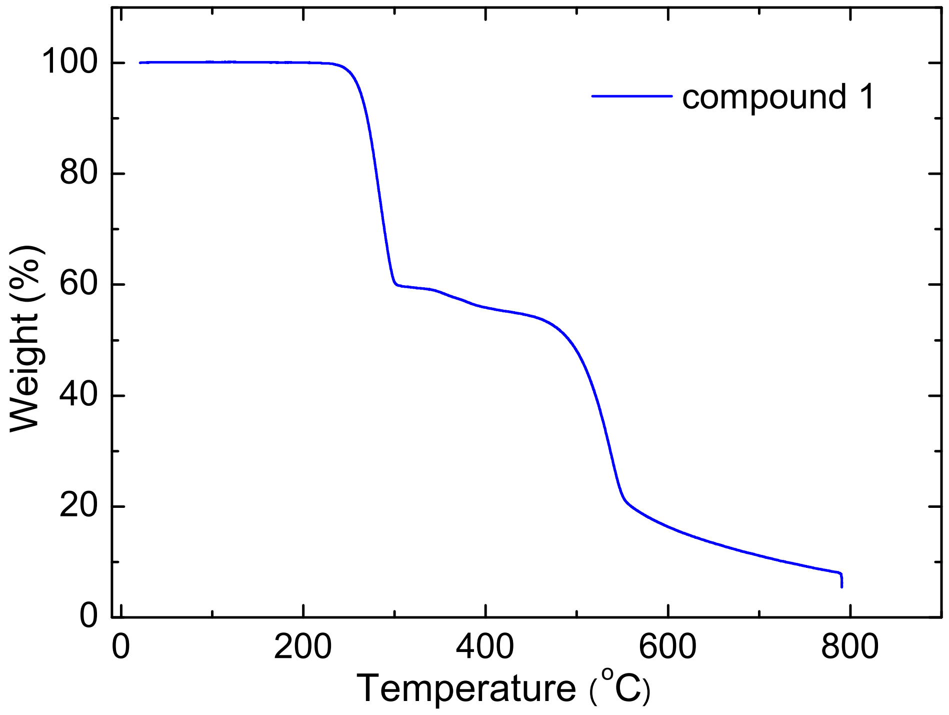

Figure S2. Thermogravimetric (TG) analysis diagram of compound **1**.

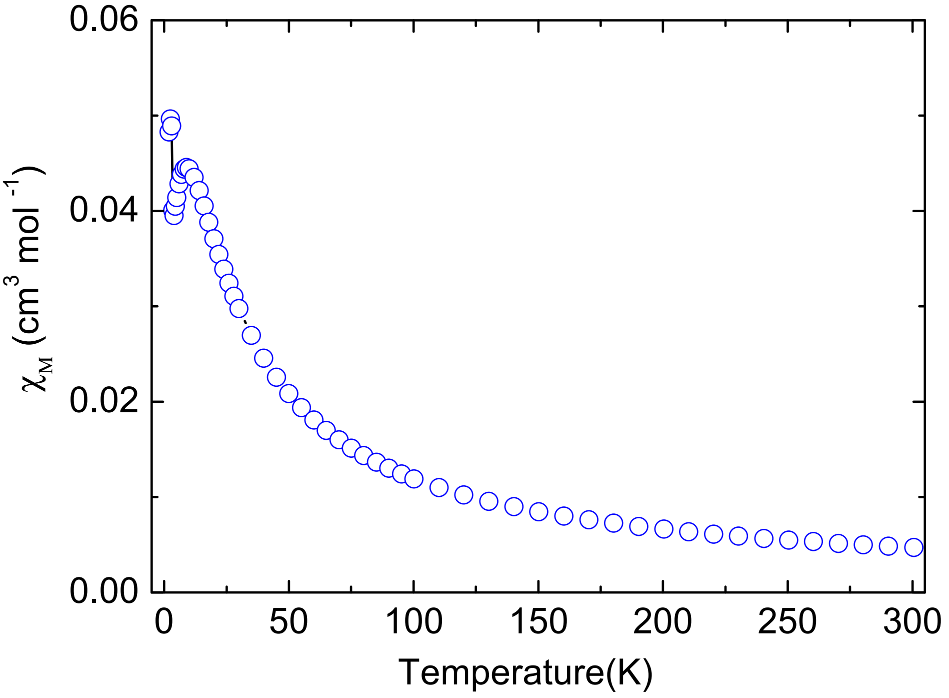

Figure S3. Plot of χ_M (○) *vs.* *T* for a powdered sample of compound **1**.

.

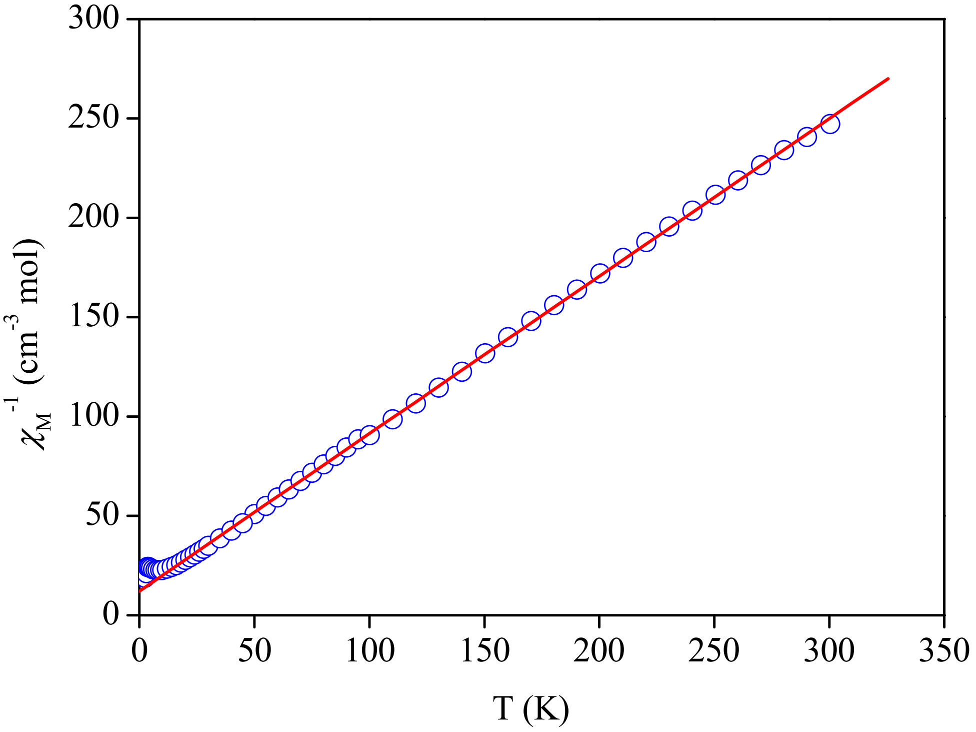

Figure S4. Plot of χ_M^{-1} (○) *vs.* T for a powdered sample of compound **1**. The solid line represents the best fit χ_M^{-1} above 50 K with a Curie–Weiss law.

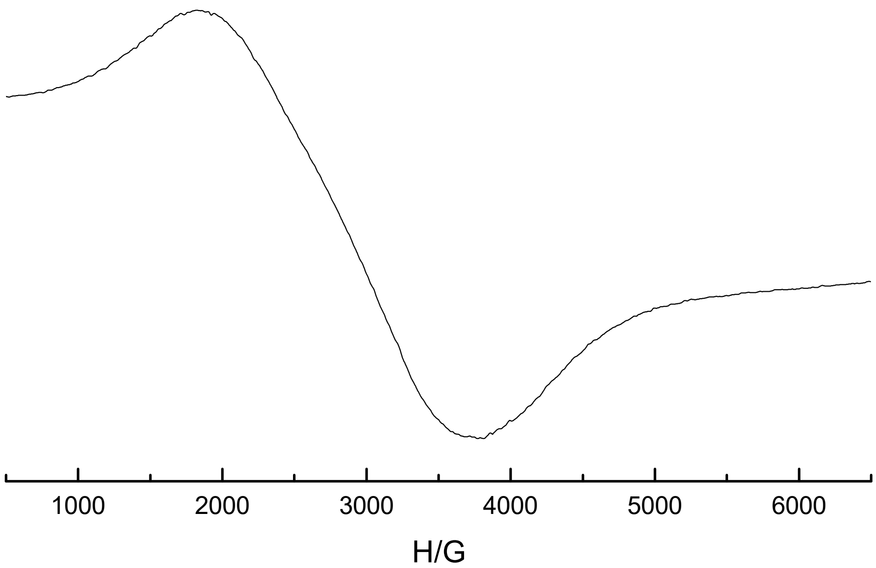

Figure S5. EPR spectra of a powdered compound **1** collected at 77 K.
